# Hierarchical Cell Death Program Disrupts the Intracellular Niche Required for Burkholderia thailandensis Pathogenesis

**DOI:** 10.1128/mBio.01059-21

**Published:** 2021-06-22

**Authors:** David E. Place, Shelbi Christgen, Shraddha Tuladhar, Peter Vogel, R. K. Subbarao Malireddi, Thirumala-Devi Kanneganti

**Affiliations:** a Department of Immunology, St. Jude Children's Research Hospital, Memphis, Tennessee, USA; b Animal Resources Center and the Veterinary Pathology Core, St. Jude Children's Research Hospital, Memphis, Tennessee, USA; Emory University School of Medicine

**Keywords:** pyroptosis, apoptosis, necroptosis, inflammasome, caspase-1, caspase-8, caspase-11, *Burkholderia thailandensis*, RIPK1, RIPK3, MLKL, PANoptosis, PANoptosome, type six secretion system, T6SS, VgrG5, virulence, cell fusion, NLRC4, NLRP3, gasdermin D, caspase-3, caspase-7, macrophage

## Abstract

*Burkholderia* infections can result in serious diseases with high mortality, such as melioidosis, and they are difficult to treat with antibiotics. Innate immunity is critical for cell-autonomous clearance of intracellular pathogens like *Burkholderia* by regulating programmed cell death. Inflammasome-dependent inflammatory cytokine release and cell death contribute to host protection against Burkholderia pseudomallei and Burkholderia thailandensis; however, the contribution of apoptosis and necroptosis to protection is not known. Here, we found that bone marrow-derived macrophages (BMDMs) lacking key components of pyroptosis died via apoptosis during infection. BMDMs lacking molecules required for pyroptosis, apoptosis, and necroptosis (PANoptosis), however, were significantly resistant to B. thailandensis*-*induced cell death until later stages of infection. Consequently, PANoptosis-deficient BMDMs failed to limit B. thailandensis*-*induced cell-cell fusion, which permits increased intercellular spread and replication compared to wild-type or pyroptosis-deficient BMDMs. Respiratory B. thailandensis infection resulted in higher mortality in PANoptosis-deficient mice than in pyroptosis-deficient mice, indicating that, in the absence of pyroptosis, apoptosis is essential for efficient control of infection *in vivo*. Together, these findings suggest both pyroptosis and apoptosis are necessary for host-mediated control of *Burkholderia* infection.

## INTRODUCTION

Infection with Burkholderia pseudomallei causes the disease melioidosis, which affects patients in Southeast Asia and Northern Australia and can infect multiple other animal species (1). Similarly, Burkholderia mallei causes glanders, a rare disease contracted from infected horses. The severe diseases caused by B. mallei and B. pseudomallei, which, if left untreated, cause mortality in roughly 50% of patients, has led to their classification as potential bioweapons (2). *Burkholderia* spp. are also inherently antibiotic resistant, which has spurred interest in studying their virulence mechanisms to develop alternative treatments for infection. Additionally, the intracellular bacterium B. thailandensis is a closely related model for the more virulent B. pseudomallei and B. mallei and is important for studying *Burkholderia* virulence mechanisms (3–5).

Innate immune signaling and inflammatory cytokines are critical for controlling *Burkholderia* infection, but how these inflammatory pathways provide protection during infection is not well understood (6, 7). Programmed cell death pathways, which are critically regulated by these innate immune pathways, have emerged as an important potential mechanism for limiting *Burkholderia* infections.

Programmed cell death is important for normal organismal development, as well as clearance of intracellular pathogens from infected cells (8). Pyroptosis is an inflammatory form of lytic cell death which is initiated by host sensing of conserved microbial ligands or cellular danger signals (9). Activation of cytosolic inflammasome sensors by these ligands leads to the activation of caspase-1, which cleaves substrates interleukin 1β (IL-1β), IL-18, and the pore-forming executioner molecule gasdermin D (GSDMD) into their active forms, thereby driving pyroptosis (10–17). GSDMD can also be activated by caspase-11-dependent sensing of intracellular lipopolysaccharide (LPS), resulting in GSDMD-dependent pyroptosis (18–21). Necroptosis is another form of lytic cell death, mediated by RIPK1, TRIF, or ZBP1-dependent activation of RIPK3, which then phosphorylates the pore-forming executioner molecule MLKL (22–26). This process largely occurs in cells where caspase-8 activity is inhibited, as caspase-8 activity limits RIPK1 and RIPK3 under homeostatic conditions (25–31). Apoptosis involves initiator caspases (caspase-8 and caspase-9), which cleave and activate executioner caspases-3, -7, and -6 that then cleave downstream substrates, resulting in the characteristic features of apoptosis, including membrane blebbing, disruption of the nucleus, and cell shrinkage (32). Phagocytes recognize and clear apoptotic cells by phagocytosis, which limits the release of inflammatory intracellular components (33), and has led to apoptosis being historically considered a minimally inflammatory cell death process. More recent studies, however, have shown that under certain conditions, cross talk between pyroptotic and apoptotic pathways can promote inflammation (34–38). In addition, necroptosis has been shown to activate pyroptosis (39). This extensive cross talk between cell death pathways has led to the development of the concept of PANoptosis, which is defined as a unique, physiologically relevant, inflammatory programmed cell death pathway activated by specific triggers and regulated by the PANoptosome complex (40–42). The PANoptosome provides a molecular scaffold for contemporaneous engagement of key shared components of the pyroptosis, apoptosis, and necroptosis pathways (23, 43–46). The ability of these molecules to interact allows for intricate coregulation between the cell death pathways that had previously been thought to be independent. PANoptosis has been implicated in infectious and autoinflammatory diseases, cancer, and beyond (23, 35, 37, 43–51), and the molecular details and phenotypic outcomes of the cross talk and coregulation among pyroptosis, apoptosis, and necroptosis are dependent on the stimulus provided.

Understanding the role of cell death pathways during *Burkholderia* infection is critical to identifying ways to counteract these pathogens. Previous studies have established an important role for pyroptosis in the control of B. thailandensis (52–58). The role of apoptosis and necroptosis in B. thailandensis infection, however, is not well understood. In this study, we identified a critical role for both pyroptosis and apoptosis in restricting B. thailandensis intracellular replication, which was significantly impaired by deletion of key components of PANoptosis. Consequently, macrophages deficient in PANoptotic molecules were unable to limit the cell-cell fusion and intercellular spread of B. thailandensis, which permitted dramatically increased bacterial replication. Loss of PANoptosis similarly rendered mice highly susceptible to B. thailandensis respiratory infection. Together, these findings suggest a key role for multiple programmed cell death pathways in restricting *Burkholderia*-induced cell-cell fusion, bacterial infection, and infection-induced mortality.

## RESULTS

### B. thailandensis infection-induced cell death is delayed but ultimately increases in *Casp8/Ripk3/Casp1/11*^−/−^ BMDMs.

**FIG 1:**
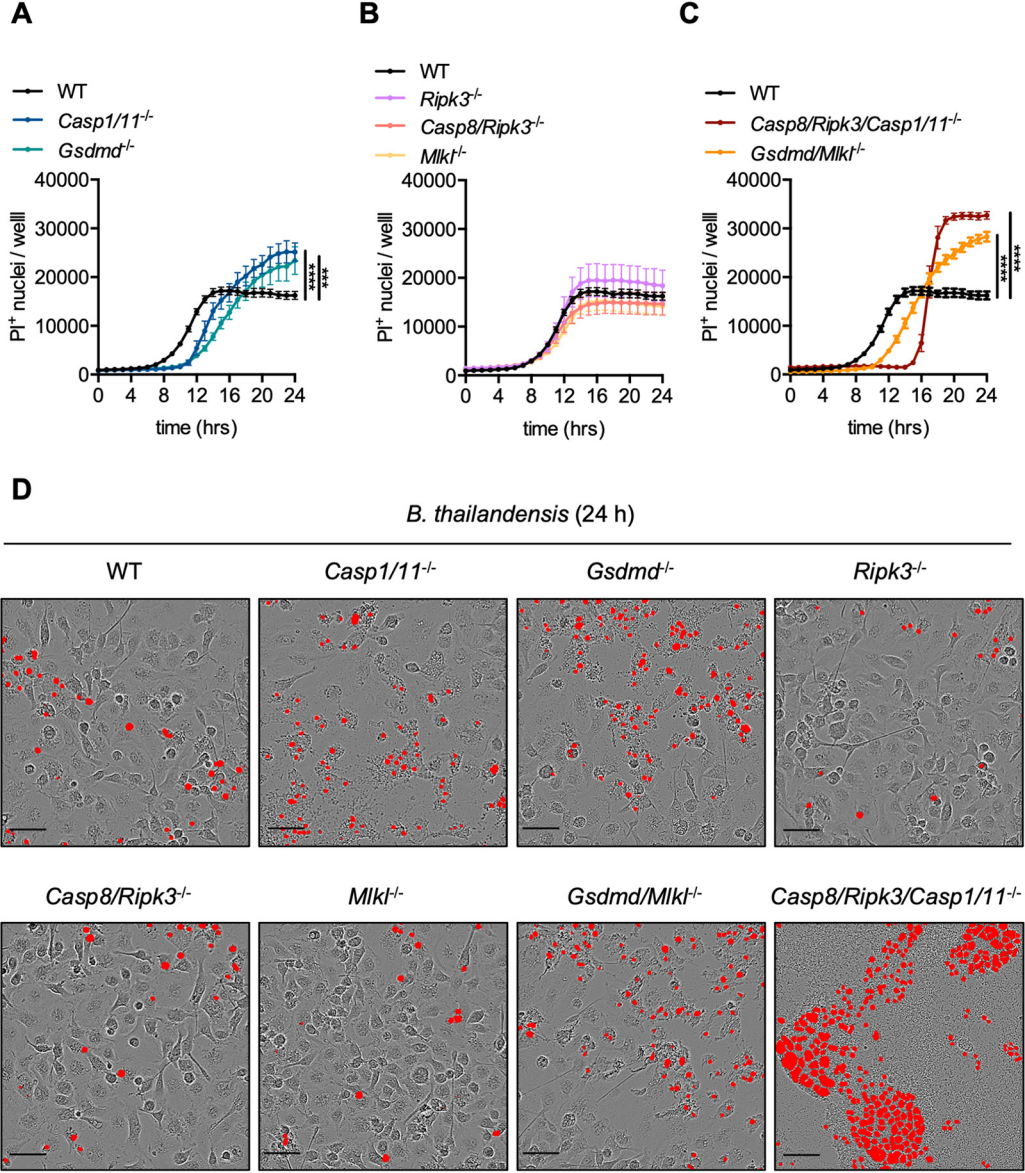
B. thailandensis infection-induced cell death is delayed but ultimately increases in *Casp8/Ripk3/Casp1/11*^−/−^ BMDMs. Bone marrow-derived macrophages (BMDMs) were infected with B. thailandensis (MOI 5) and cell death was tracked in the indicated WT or knockout cells deficient in components of pyroptosis (*Casp1/11*^−/−^, *Gsdmd*^−/−^), necroptosis (*Ripk3*^−/−^, *Mlkl*^−/−^), apoptosis/necroptosis (*Casp8/Ripk3*^−/−^), pyroptosis/necroptosis (*Gsdmd/Mlkl*^−/−^), or PANoptosis (*Casp8/Ripk3/Casp1/11*^−/−^). (A to C) Quantification of propidium iodide (PI)^+^ nuclei over 24 h of infection in the indicated BMDM genotypes with (D) representative images from 24 h postinfection showing PI^+^ nuclei (red object mask). Scale bars (black) indicate 50 μm. Significance was determined by two-way ANOVA with Tukey’s multiple-comparison test: *, ≤0.05; **, ≤0.01; ***, ≤0.001; ****, ≤0.0001; n.s., not significant. Data are representative of one of at least three independent biological replicate experiments where at least four replicate wells, with nine images per well, were quantified to determine the average nuclei count per well.

Macrophages infected with B. thailandensis undergo NLRP3-, NLRC4-, caspase-1-, caspase-11-, and GSDMD-dependent pyroptosis (52–60), but the role of other cell death pathways during infection is poorly understood. We therefore assessed B. thailandensis infection in BMDMs that lacked critical components of cell death pathways. Deletion of caspase-8 from mice results in embryonic lethality due to spontaneous activation of necroptosis mediated by RIPK3 and MLKL; this lethality can be rescued by concurrent deletion of caspase-8 and either RIPK3 or MLKL (61, 62). *Casp8/Ripk3*^−/−^ mice can then be used to study the combined role of the apoptotic and necroptotic pathways during infection with B. thailandensis. Additionally, *Casp8/Ripk3/Casp1/11*^−/−^ mice, which are deficient in PANoptosis, allow the study of the combined roles of pyroptosis, caspase-8-dependent apoptosis, and necroptosis during infection (43, 49). To determine the contribution of pyroptosis, apoptosis, and necroptosis, BMDMs deficient in key components of pyroptosis (*Casp1/11*^−/−^ or *Gsdmd*^−/−^), apoptosis/necroptosis (*Casp8/Ripk3*^−/−^), necroptosis (*Ripk3*^−/−^ or *Mlkl*^−/−^), pyroptosis/necroptosis (*Gsdmd/Mlkl*^−/−^), or PANoptosis (*Casp8/Ripk3/Casp1/11*^−/−^) were infected with B. thailandensis. At 24 h postinfection, there was no difference between cell death in WT cells and cells deficient in necroptosis, a moderate increase in cell death in pyroptosis-deficient cells, and a further increased number of dead PANoptosis-deficient cells (Fig. 1A to D). The kinetics of cell death suggest that pyroptosis-competent BMDMs (WT, *Ripk3*^−/−^, *Casp8/Ripk3*^−/−^, and *Mlkl*^−/−^) rapidly undergo pyroptosis (Fig. 1B). The moderate increase in cell death observed in cells lacking pyroptosis molecules (GSDMD and caspase-1/11) at later time points is likely due to a delay in cell death, which permits increased time for B. thailandensis-induced intercellular spread via cell-cell fusion (Fig. 1A and D). The cell death observed in BMDMs lacking key components of pyroptosis also suggested that additional cell death pathways are secondarily activated by B. thailandensis infection. Consistent with this observation, PANoptosis-deficient BMDMs undergo further delayed cell death compared with both WT cells and BMDMs lacking key pyroptosis machinery (GSDMD or caspase-1/11) (Fig. 1A, C, and D). Together, these findings suggest hierarchical activation of pyroptosis and apoptosis is required for activation of macrophage cell death during B. thailandensis infection. We further observed that BMDMs deficient in key components of PANoptosis formed significantly larger multinucleated giant cells (MNGCs) compared to infected WT BMDMs (Fig. 1D, Video S1 and S2 in the supplemental material). Together, these data suggest that macrophages utilize both pyroptosis and apoptosis cell death pathways to limit B. thailandensis infection.

### B. thailandensis infection induces pyroptotic and apoptotic cell death in BMDMs.

**FIG 2:**
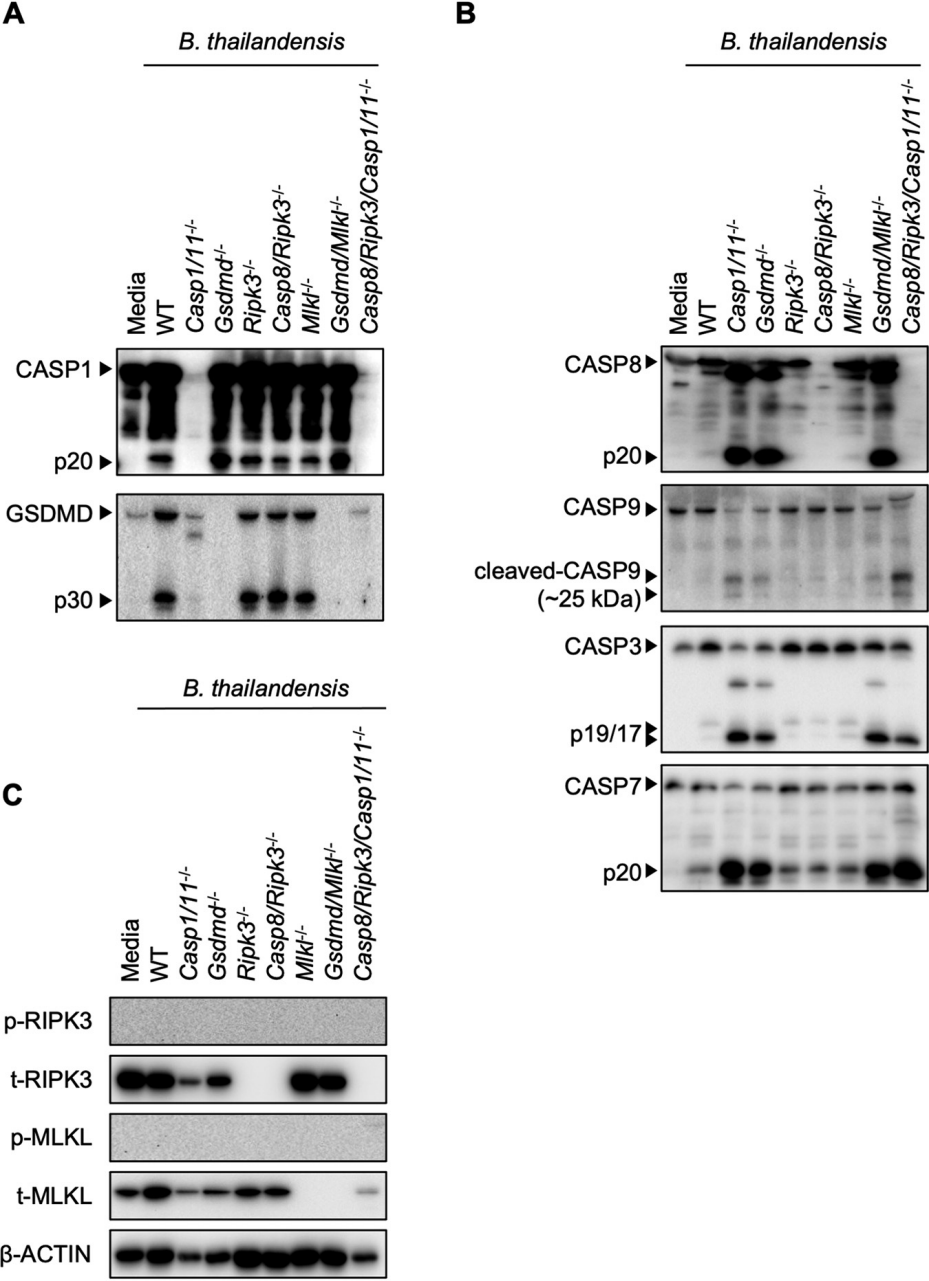
B. thailandensis infection induces pyroptotic and apoptotic cell death in BMDMs. (A to C) Bone marrow-derived macrophages (BMDMs) were infected with B. thailandensis and combined cell and supernatant lysates (for caspase and gasdermin D [GSDMD] blots) or cell lysates were collected at 24 h postinfection. Immunoblots were performed to detect pyroptosis activation, indicated by cleavage of caspase-1 (CASP1) and GSDMD (A), apoptosis activation, indicated by cleavage of caspase-8 (CASP8), caspase-9 (CASP9), caspase-3 (CASP3), and caspase-7 (CASP7) (B), or necroptosis activation, indicated by phosphorylation of RIPK3 (p-RIPK3) and MLKL (p-MLKL) (C). Data are representative of one of at least three independent biological experiments, and BMDMs used to generate data in Fig. 2 were also used to generate data presented in Fig. S1 in the supplemental material and Fig. 3B to D.

Given our observation that PANoptosis-deficient BMDMs (*Casp8/Ripk3/Casp1/11^−/−^*) displayed distinct cell death activation kinetics during B. thailandensis infection, we biochemically examined the activation of pyroptotic, apoptotic, and necroptotic pathways at 24 h of infection. Consistent with previous studies (53, 55–57, 59, 60), B. thailandensis infection in WT BMDMs activated caspase-1 and GSDMD (Fig. 2A, Fig. S1A). In B. thailandensis*-*infected WT BMDMs, apoptotic caspases were also slightly activated (Fig. 2B, Fig. S1B), suggesting apoptotic pathways may also regulate cell death during infection. In pyroptosis-deficient *Casp1/11*^−/−^ or *Gsdmd*^−/−^ BMDMs, caspase-8 and caspases-3/7 were activated to a greater extent than in WT, suggesting that macrophages lacking the machinery for pyroptosis can compensate by activating apoptosis to kill infected macrophages (Fig. 2B). Our data indicated that BMDMs lacking necroptosis components (RIPK3 or MLKL) underwent similar cell death compared with WT BMDMs (Fig. 1B and D), which is consistent with the observed activation of caspase-1, GSDMD, and apoptotic caspases in these cells (Fig. 2A and B). Phosphorylation of RIPK3 and MLKL was not observed during infection at the multiplicity of infection (MOI) and time points used (Fig. 2C, Fig. S1C), suggesting necroptosis is not induced by B. thailandensis under these conditions. Cell death in pyroptosis-deficient macrophages also reduced the amount of total protein compared to WT, likely via apoptotic caspase-mediated cleavage (Fig. 2C).

10.1128/mBio.01059-21.1 FIG S1 Apoptosis is activated late during infection in *Casp8/Ripk3/Casp1/11*^−/−^ BMDMs. Bone marrow-derived macrophages (BMDMs) were infected with B. thailandensis and combined cell and supernatant lysates (for caspase and gasdermin D [GSDMD] blots) or cell lysates were collected at the indicated time-points. (A to C) Immunoblots were performed to detect pyroptosis activation, indicated by cleavage of caspase-1 (CASP1) and GSDMD (A), apoptosis activation, indicated by cleavage of caspase-8 (CASP8), caspase-9 (CASP9), caspase-3 (CASP3), and caspase-7 (CASP7) (B), or necroptosis activation, indicated by phosphorylation of RIPK3 (p-RIPK3) and MLKL (p-MLKL) (C). Data are representative of at least three independent biological experiments, and BMDMs used to generate data in Fig. S1 were also used to generate data presented in Fig. 2. Download FIG S1, PDF file, 0.1 MB. Copyright © 2021 Place et al. 2021 Place et al. https://creativecommons.org/licenses/by/4.0/ This content is distributed under the terms of the Creative Commons Attribution 4.0 International license.

While *Casp8/Ripk3/Casp1/11^−/−^* BMDMs were protected from cell death in the early stage of infection with B. thailandensis, this delay in cell death resulted in increased formation of MNGCs via cell-cell fusion (Fig. 1D). At later stages of infection, these MNGCs underwent focal, synchronized cell death (Video S1 and S2). Consistent with this finding, the initiator caspase-9 and executioner caspases-3/7 were activated in *Casp8/Ripk3/Casp1/11^−/−^* BMDMs at 24 h postinfection (Fig. 2B, Fig. S1B), suggesting intrinsic apoptosis is ultimately activated in these MNGCs. Together, these data indicate that pyroptosis and caspase-8-dependent apoptosis predominantly initiate rapid cell death during B. thailandensis infection to limit the bacterial intracellular niche.

10.1128/mBio.01059-21.5 VIDEO S1 Time-lapse video of B. thailandensis infection in WT BMDMs. Bone marrow-derived macrophages (BMDMs) were infected with B. thailandensis (MOI 5), and images were collected hourly on an IncuCyte S3 (propidium iodide [PI]^+^ nuclei with red analysis mask). Download VIDEO S1, AVI file, 9.3 MB. Copyright © 2021 Place et al. 2021 Place et al. https://creativecommons.org/licenses/by/4.0/ This content is distributed under the terms of the Creative Commons Attribution 4.0 International license.

10.1128/mBio.01059-21.6 VIDEO S2 Time-lapse video of B. thailandensis infection in *Casp8/Ripk3/Casp1/11*^−/−^ BMDMs. Bone marrow-derived macrophages (BMDMs) were infected with B. thailandensis (MOI 5) and images were collected hourly on an IncuCyte S3 (propidium iodide [PI]^+^ nuclei with red analysis mask). Download VIDEO S2, AVI file, 10.9 MB. Copyright © 2021 Place et al. 2021 Place et al. https://creativecommons.org/licenses/by/4.0/ This content is distributed under the terms of the Creative Commons Attribution 4.0 International license.

### *Casp8/Ripk3/Casp1/11*^−/−^ BMDMs fail to restrict B. thailandensis cell-cell fusion and replication.

**FIG 3:**
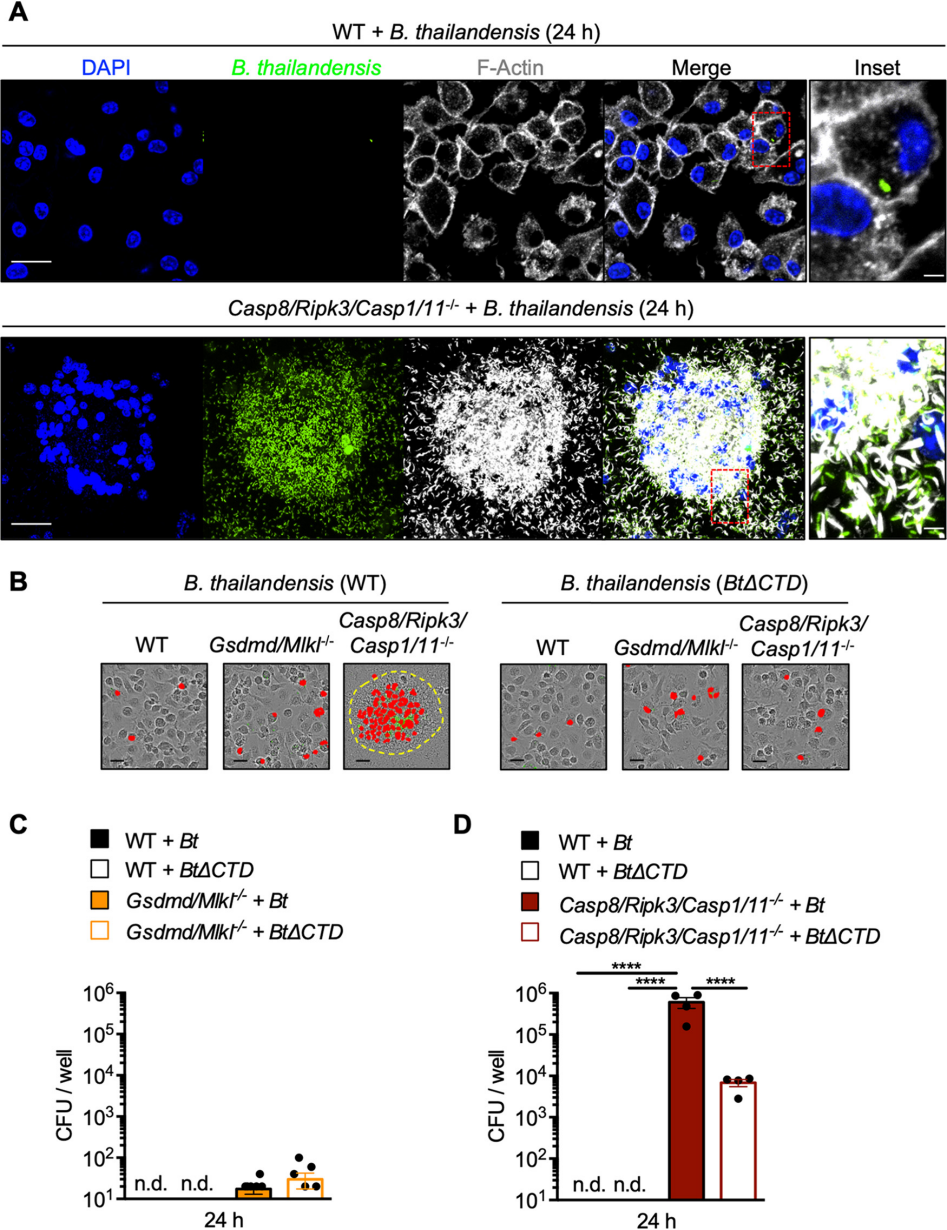
*Casp8/Ripk3/Casp1/11*^−/−^ BMDMs fail to restrict B. thailandensis cell-cell fusion and replication. Bone marrow-derived macrophages (BMDMs) were infected with B. thailandensis (GFP^+^) and fixed at 24 h postinfection. (A) The indicated BMDMs were stained for F-actin (white) and nuclei (blue), and bacteria were visualized by bacterial green fluorescent protein (GFP) expression (green); images were collected by confocal microscopy. (B) Representative images of B. thailandensis-infected BMDMs at 24 h postinfection with SYTOX Green-stained nuclei (red). (C and D) Intracellular CFU at 24 h postinfection were quantified from WT (*n* = 4) and pyroptosis/necroptosis-deficient (*Gsdmd/Mlkl*^−/−^) BMDMs (*n* = 8) (C) or PANoptosis-deficient (*Casp8/Ripk3/Casp1/11*^−/−^) BMDMs (*n* = 4) infected with WT B. thailandensis (*Bt*) or the *vgrG5*Δ*CTD* mutant (*Bt*Δ*CTD*) (D). Scale bars indicate (white) 20 μm, (white, inset) 3.3 μm, or (black) 25 μm. Yellow-dotted line (B) indicates the boundary of multinucleated giant cells (MNGCs). Significance was determined by one-way ANOVA with Holm-Sidak’s multiple-comparison test: *, ≤0.05; **, ≤0.01; ***, ≤0.001; ****, ≤0.0001; n.d., not detected. Data are representative of at least two independent biological experiments, and BMDMs used to generate data in Fig. 3B to D were also used to generate data presented in Fig. 2.

The significantly delayed cell death and extensive cell-cell fusion observed in B. thailandensis-infected *Casp8/Ripk3/Casp1/11^−/−^* BMDMs suggested that bacterial infection is poorly controlled in these cells compared to WT. The physiological importance of cell-cell fusion in the infectious process of B. thailandensis, B. pseudomallei, B. mallei, and Burkholderia oklahomensis is still poorly understood, but is thought to contribute to intercellular spread and promote bacterial replication. To determine the impact of deleting key PANoptosis components, we first examined cell-cell fusion and the bacterial burden of BMDMs by confocal microscopy. As expected, cell-cell fusion and the intracellular bacterial burden were notably increased in *Casp8/Ripk3/Casp1/11^−/−^* BMDMs compared to WT (Fig. 3A), indicating that delayed cell death in *Casp8/Ripk3/Casp1/11^−/−^* BMDMs allows for increased cell-cell fusion and B. thailandensis intercellular spread to neighboring cells.

B. thailandensis, B. pseudomallei, B. mallei, and *B. oklahomensis* possess a unique type six secretion system (T6SS) effector protein, VgrG5, which is required for infection-induced host cell-cell fusion, formation of MNGCs, and intercellular spread (63–66). To distinguish between the role of bacterial-mediated cell-cell fusion and host-mediated cell death in regulating bacterial replication, we utilized WT B. thailandensis and a mutant strain of B. thailandensis *vgrG5*Δ*CTD* (indicated as *Bt*Δ*CTD*) lacking the C-terminal domain of VgrG5 that is required for inducing eukaryotic cell-cell fusion (65). We first determined that cell-cell fusion in B. thailandensis*-*infected *Casp8/Ripk3/Casp1/11^−/−^* BMDMs required VgrG5 (Fig. 3B). To quantify intracellular bacterial replication, BMDMs lacking executioners of pyroptosis/necroptosis (*Gsdmd/Mlkl*^−/−^) or PANoptosis (*Casp8/Ripk3/Casp1/11^−/−^*) were infected, and intracellular bacterial CFU counts were determined after 24 h. BMDMs deficient in the pyroptosis and necroptosis executioners GSDMD and MLKL were largely able to restrict intracellular bacterial replication, suggesting that apoptosis limited the intracellular replication of B. thailandensis (Fig. 3B and C). In contrast, significantly increased bacterial replication was observed in *Casp8/Ripk3/Casp1/11^−/−^* BMDMs compared to WT (Fig. 3B and D). These findings indicate that the delayed cell death observed in *Casp8/Ripk3/Casp1/11^−/−^* BMDMs results in failure to restrict intracellular replication and cell-to-cell spread of B. thailandensis (Fig. 3B and D). B. thailandensis lacking VgrG5 cell-cell fusion activity also replicated intracellularly to a higher degree in *Casp8/Ripk3/Casp1/11^−/−^* BMDMs than in WT (Fig. 3D), but loss of cell-cell fusion limited the overall replicative capacity, reinforcing the importance of cell-cell fusion and intercellular spread to the pathogenesis of the B. thailandensis. These data, together with the observation that pyroptosis is the primary cell death pathway activated in WT BMDMs (Fig. 2), suggest that intracellular replication and spread of B. thailandensis is restricted by pyroptosis but, in the absence of pyroptosis, caspase-8-dependent apoptosis compensates to mediate intracellular clearance.

### *Casp8/Ripk3/Casp1/11*^−/−^ mice fail to limit respiratory B. thailandensis infection.

**FIG 4:**
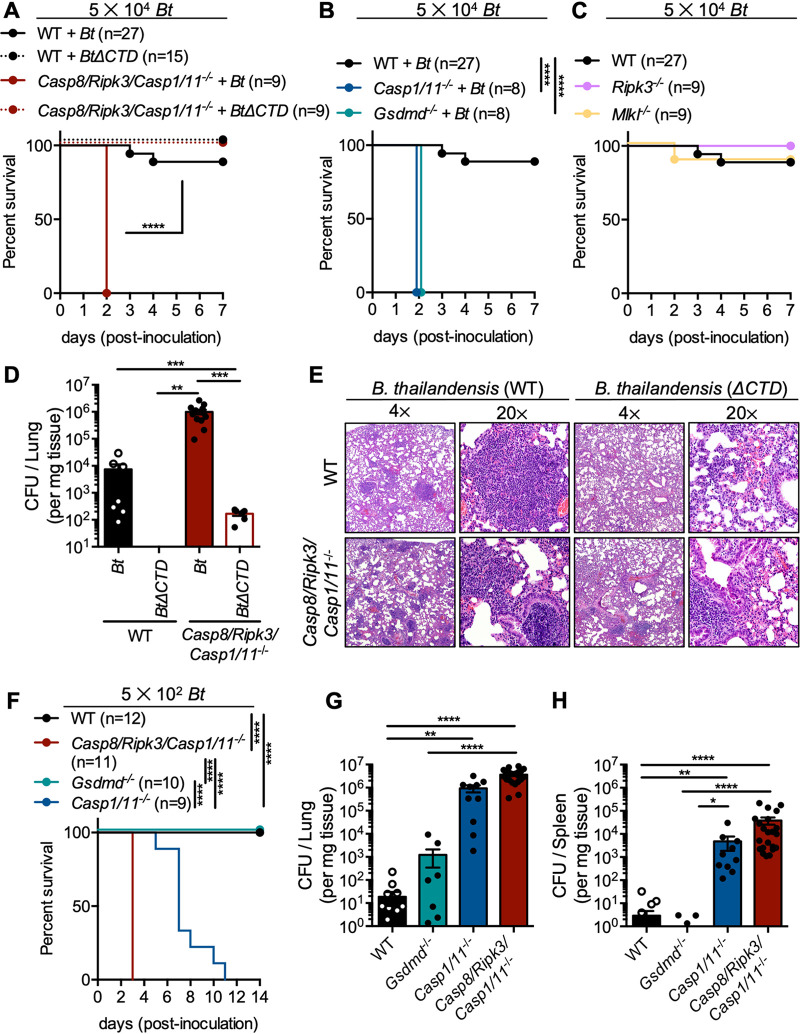
*Casp8/Ripk3/Casp1/11*^−/−^ mice fail to limit respiratory B. thailandensis infection. Mice were intranasally infected with WT (*Bt*) or *vgrG5*Δ*CTD* (indicated as *Bt*Δ*CTD)* B. thailandensis. (A to C) Survival of infected mice (5 × 10^4^ dose) was monitored in WT and PANoptosis-deficient *Casp8/Ripk3/Casp1/11*^−/−^ (A), pyroptosis-deficient (*Casp1/11*^−/−^ or *Gsdmd*^−/−^) (B), or necroptosis-deficient (*Ripk3*^−/−^ or *Mlkl*^−/−^) mice (C). (D) At day 2 postinoculation, CFU counts were determined from lungs of infected mice (WT + *Bt* [*n* = 7]; WT + *Bt*Δ*CTD* [*n* = 5]; *Casp8/Ripk3/Casp1/11*^−/−^ + *Bt* [*n* = 13]; *Casp8/Ripk3/Casp1/11*^−/−^ + *Bt*Δ*CTD* [*n* = 6]). (E) Representative histological images from infected (5 × 10^4^ dose) lungs at day 2 postinoculation, at 4× and 20× magnification, of each condition are shown and were collected by a veterinary pathologist (P.V.). (F) Survival of infected mice (5 × 10^2^ dose) was monitored. (G and H) At day 3 postinoculation, CFU counts were determined from lungs (G) or spleens (H) of infected mice (WT + *Bt*, [*n* = 20]; *Gsdmd*^−/−^ + *Bt* [*n* = 11]; *Casp1/11*^−/−^ + *Bt* [*n* = 10]; *Casp8/Ripk3/Casp1/11*^−/−^ + *Bt* [*n* = 22]). Significance was determined by the log rank test (A to C, F), one-way ANOVA with Holm-Sidak’s multiple-comparison test (D), or one-way ANOVA with Dunn’s multiple-comparison test (G and H): *, ≤0.05; **, ≤0.01; ***, ≤0.001; ****, ≤0.0001, ND, not detected. Data are pooled from at least two independent experiments (A to D), one experiment (E), or at least two experiments (G and H).

The failure of *Casp8/Ripk3/Casp1/11^−/−^* BMDMs to restrict B. thailandensis intercellular spread via cell-cell fusion and intracellular replication suggested that *Casp8/Ripk3/Casp1/11^−/−^* mice would be highly susceptible to severe lethal infection. To determine whether PANoptosis-deficient mice are more susceptible to lethality following B. thailandensis infection, mice were inoculated intranasally with 5 × 10^4^ B. thailandensis. *Casp8/Ripk3/Casp1/11^−/−^* mice were significantly more susceptible to lethal infection with WT B. thailandensis than were WT mice (Fig. 4A). Previous studies have found that B. thailandensis *vgrG5*Δ*CTD* infection is attenuated compared to WT B. thailandensis in WT mice (59, 65), but whether cell-cell fusion is important for virulence in the absence of cell death is unknown. Because B. thailandensis *vgrG5*Δ*CTD* was not efficiently cleared from *Casp8/Ripk3/Casp1/11^−/−^* BMDMs (Fig. 3D), we hypothesized that *Casp8/Ripk3/Casp1/11^−/−^* mice may be more susceptible to infection than WT mice. Contrary to our hypothesis, *Casp8/Ripk3/Casp1/11^−/−^* mice infected with B. thailandensis *vgrG5*Δ*CTD* survived challenge, suggesting that bacterial-mediated cell-cell fusion is necessary for full virulence and that loss of cell-cell fusion may result in a self-limiting infection, even in the absence of PANoptosis (Fig. 4A). Consistent with previous studies, caspase-1/11 and GSDMD were required for host protection against infection at this infectious dose (Fig. 4B) (58). Similar to the phenotype observed in *Casp8/Ripk3/Casp1/11^−/−^* mice, B. thailandensis *vgrG5*Δ*CTD* infection was also attenuated in mice lacking caspase-1/11 or GSDMD (Fig. S2A). Mice lacking necroptosis components RIPK3 or MLKL survived similarly to WT mice during infection with B. thailandensis, suggesting necroptosis is not required to control B. thailandensis (Fig. 4C). Mice lacking both GSDMD and MLKL (pyroptosis/necroptosis) were similarly susceptible to lethal infection as *Gsdmd*^−/−^ mice (Fig. S2B). Mice lacking caspase-8, RIPK3, and RIPK1 (apoptosis/necroptosis) survived infection similarly to mice lacking either RIPK3 or MLKL, suggesting pyroptosis primarily protects against B. thailandensis in pyroptosis-competent mice (Fig. S2C). To determine whether bacterial replication was regulated in mice similarly to what we observed in BMDMs (Fig. 3C and D), bacterial CFU counts were determined in lung tissues collected at day 2 postinoculation. Consistent with the increased bacterial replication observed in *Casp8/Ripk3/Casp1/11^−/−^* BMDMs, *Casp8/Ripk3/Casp1/11^−/−^* mice permitted increased replication of B. thailandensis compared to WT mice (Fig. 4D). A trend toward increased bacterial burden was also observed during infection with the B. thailandensis *vgrG5*Δ*CTD* strain in PANoptosis-deficient mice compared to WT mice (Fig. 4D). Consistent with the mouse survival data, *Casp8/Ripk3/Casp1/11^−/−^* mice, which succumbed to WT B. thailandensis infection, exhibited increased lung tissue damage at day 2 postinoculation (Fig. 4E, Fig. S3A to E). Notably, *Casp8/Ripk3/Casp1/11^−/−^* mice had significantly increased accumulations of degenerating/necrotic polymorphonuclear leukocytes (PMNs) that plugged terminal airways and were associated with necrosis of pulmonary parenchyma. In contrast, pulmonary lesions in WT mice infected with WT B. thailandensis were smaller, more clearly defined, and consisted of accumulations of intact PMNs and macrophages, with no blocking of terminal airways or notable damage to alveolar walls (Fig. S3A to E).

10.1128/mBio.01059-21.2 FIG S2 Additional survival data for B. thailandensis infection. Mice were intranasally infected with WT (*Bt*) or *vgrG5*Δ*CTD* (indicated as *Bt*Δ*CTD)* B. thailandensis. Survival of infected mice (5 × 10^4^ dose) was monitored in pyroptosis-deficient (*Casp1/11*^−/−^ or *Gsdmd*^−/−^) (A), pyroptosis/necroptosis-deficient (*Gsdmd/Mlkl*^−/−^) (B), or apoptosis/necroptosis-deficient (*Casp8/Ripk3/Ripk1*^−/−^) (C) mice. Survival data relate to and include data presented in Fig. 4A to C. Mouse survival data are pooled from at least two independent experiments. Significance was determined by the log rank test: *, ≤0.05; **, ≤0.01; ***, ≤0.001; ****, ≤0.0001. Download FIG S2, PDF file, 0.1 MB. Copyright © 2021 Place et al. 2021 Place et al. https://creativecommons.org/licenses/by/4.0/ This content is distributed under the terms of the Creative Commons Attribution 4.0 International license.

10.1128/mBio.01059-21.3 FIG S3 Histopathology quantification for B. thailandensis-infected lungs. Fixed lung sections from mice infected with WT B. thailandensis (*Bt*) or the *vgrG5*Δ*CTD* mutant (*Bt*Δ*CTD*) (5 × 10^4^ dose each) stained with hematoxylin and eosin were scored (as described in the Materials and Methods) in a blinded fashion by a trained veterinary pathologist (P.V.). (A to E) Interstitial inflammation, macrophages (A), interstitial inflammation, lymphocytes (B), intra-alveolar neutrophils (C), intra-alveolar macrophages (D), and tissue necrosis and degenerating neutrophils (polymorphonuclear leukocytes [PMNs]) (E) scores are presented. Data relate to the representative images in Fig. 4E. Pathology scores were determined from a single experiment. Significance was determined by one-way ANOVA with Tukey’s multiple-comparison test: *, ≤0.05; **, ≤0.01; ***, ≤0.001; ****, ≤0.0001. Download FIG S3, PDF file, 0.1 MB. Copyright © 2021 Place et al. 2021 Place et al. https://creativecommons.org/licenses/by/4.0/ This content is distributed under the terms of the Creative Commons Attribution 4.0 International license.

To determine whether PANoptosis-deficient mice were more susceptible to lethal infection than pyroptosis-deficient mice, mice were challenged with a lower dose of B. thailandensis (500 CFU) and monitored daily. Mice lacking GSDMD survived infection similarly to WT mice (Fig. 4F). Mice lacking the pyroptotic caspases-1/11 were more susceptible to lethal B. thailandensis infection than both WT and *Gsdmd*^−/−^ mice (Fig. 4F). However, mice deficient in PANoptosis were more susceptible to lethal infection than either *Gsdmd*^−/−^ or *Casp1/11*^−/−^ mice, suggesting that pyroptosis and apoptosis both mediate protection during B. thailandensis infection (Fig. 4F). These data also suggest that caspase-1/11 activity, independent from GSDMD cleavage, is important for limiting infection, likely due to cleavage of additional host proteins. PANoptosis-deficient mice also failed to control B. thailandensis bacterial replication in the lung and spleen during infection (Fig. 4G and H). Consistent with mouse survival during infection, WT and *Gsdmd*^−/−^ mice harbored less B. thailandensis, while *Casp1/11*^−/−^ and *Casp8/Ripk3/Casp1/11*^−/−^ mice harbored increased bacteria. Together, these data highlight the important role both pyroptosis and apoptosis play in restricting respiratory infection by B. thailandensis and further our understanding of the physiological role of *Burkholderia*-mediated cell-cell fusion in infection.

## DISCUSSION

**FIG 5:**
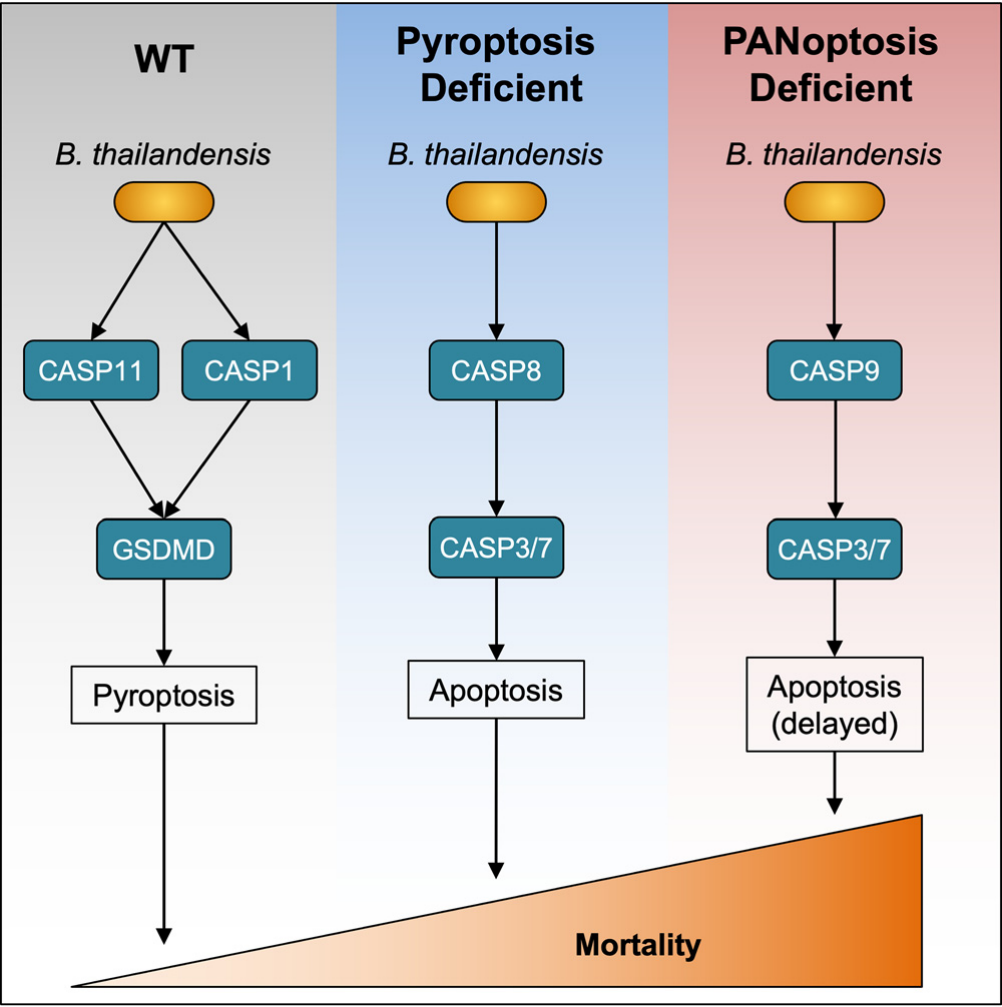
Graphical model for the roles of cell death pathways in B. thailandensis infection. The schematic summarizes the activation of cell death pathways in WT BMDMs or in knockouts of key components of pyroptosis (GSDMD or caspase-1/11) or PANoptosis (caspase-1/11, caspase-8, RIPK3) and the relative contribution of these cell death pathways during respiratory infection in mice.

In this study, we found that pyroptosis and apoptosis both regulate cell death during intracellular infection by B. thailandensis (Fig. 5). These programmed cell death pathways are necessary for the host to restrict the intracellular replication of B. thailandensis. Pyroptosis is largely responsible for promoting intracellular clearance of B. thailandensis in WT BMDMs, but in BMDMs lacking critical pyroptotic machinery, cells undergo robust apoptotic cell death. Previous studies identified a critical role for pyroptotic cell death molecules in restricting B. thailandensis but the role of apoptotic caspases and necroptosis has not been examined (52–58). Pyroptosis alone carries out multiple distinct functions in mediating protection during *Burkholderia* infections. In lung epithelial cells, caspase-11 is required for host protection, while macrophages require caspase-1, suggesting important tissue-specific roles for cell death (60). In mice, caspase-1-dependent pyroptosis is required for the production of the cytokines IL-18 and IL-1β. IL-18-dependent production of gamma interferon (IFN-γ) in mice is required for the control of B. thailandensis and B. pseudomallei, while the release of IL-1β is responsible for lethal inflammatory pathology (52, 54), indicating that cell death must be carefully balanced by the host during *Burkholderia* infection.

Here, we found that macrophages lacking key components of PANoptosis were resistant to infection-induced cell death until a later stage of B. thailandensis infection, resulting in extensive cell-cell fusion, MNGC formation, and increased intracellular bacterial replication. In the later stages of infection, macrophages underwent caspase-8-independent apoptosis characterized by activation of caspase-9 and caspase-3/7, suggesting intrinsic apoptosis can also be activated during B. thailandensis infection, consistent with previous reports (53). As a result of the defective activation of cell death in BMDMs lacking components of PANoptosis, intracellular B. thailandensis are able to persist for an extended period of time, allowing for increased infection-induced cell-cell fusion. The dramatic increase in cell-cell fusion in these cells is likely a result of the extended cell survival time, which increases the amount of time for intracellular bacteria to both replicate and form protrusions that increase contacts between cell membranes necessary for cell-cell fusion. These data also suggest that the eventual activation of caspase-9-dependent intrinsic apoptosis is insufficient to mediate protection from infection. These findings imply that infected macrophages are highly adaptable in activating programmed cell death pathways to clear intracellular pathogens, but also highlight the important role that pyroptosis and caspase-8-dependent apoptosis play in rapidly limiting B. thailandensis replication. In addition to the role cell death plays in controlling *Burkholderia* infection, questions remain as to why B. thailandensis, B. pseudomallei, B. mallei, and *B. oklahomensis* possess a unique VgrG5 C-terminal domain found only in *Burkholderia* spp., which is required for cell-cell fusion and virulence (64–67). Our data suggest that this unique virulence strategy is required to counter host-mediated restriction of bacterial intracellular replication.

Previous work has identified a critical role for cross talk between cell death pathways in regulating cell death during bacterial and viral infections with Salmonella enterica serovar Typhimurium, Listeria monocytogenes, influenza A virus (IAV), and vesicular stomatitis virus (VSV) (43). The involvement of pyroptosis, apoptosis, and necroptosis acting together established the concept of PANoptosis. Initial studies identified that activation of ZBP1-dependent PANoptosis is important for the control of IAV infection (23, 68). Similarly, RIPK1-dependent PANoptosis was identified in macrophages lacking TAK1, which undergo spontaneous PANoptosis (45, 48). Other studies found that Yersinia pestis, which secretes the T3SS effector protein YopJ, inhibits TAK1-dependent inflammatory signaling and consequently results in TAK1 inhibition-mediated inflammatory cell death (69, 70). Additionally, Shigella flexneri utilizes T3SS effectors OspC1 and OspD3 to inhibit caspase-8 and RIPK1/RIPK3, respectively (71), further showing how bacterial pathogens can modulate cell death effectors. The evolutionary arms race between host cell death pathways and pathogen-mediated inhibition of various cell death components highlights the importance of studying the molecular host-pathogen interactions of multiple pathogens (40). While B. thailandensis predominantly activated pyroptosis and apoptosis during infection, it may possess a virulence factor that inhibits necroptosis, and other *Burkholderia* species may have unique abilities to modulate these programmed cell death pathways. Inflammatory cytokines or cell-mediated immunity may also change the cell death pathways engaged in B. thailandensis-infected cells. Furthermore, dysfunction of these cell death responses may be important to permit the chronic infections sometimes observed during B. pseudomallei infection in humans (72). Overall, these studies suggest a critical role for cell death and PANoptosis in limiting infection by many distinct microbial pathogens and highlight the role that pathogens themselves can play in dictating the cell death outcome (40, 42, 43).

Using genetic mouse models deficient in key components of individual or multiple cell death pathways, we have now established a critical role for pyroptosis and apoptosis in controlling B. thailandensis infection (Fig. S4). Loss of these pathways rendered macrophages and mice highly susceptible to lethal infection by permitting unrestricted intracellular bacterial replication and intercellular spread by cell-cell fusion. Understanding these processes in the context of infection by fusogenic B. thailandensis, B. pseudomallei, B. mallei, and *B. oklahomensis* may have important implications for treating infections caused by these bacteria. The roles for programmed cell death in mediating protection against the more evolutionarily distant, nonfusogenic *Burkholderia* spp., including Burkholderia cepacia and Burkholderia cenocepacia, are likely distinct. Understanding the host immune response and virulence mechanisms of *Burkholderia* spp. is also necessary to improve treatment strategies, because *Burkholderia* spp. are inherently highly antibiotic resistant. Our data suggest more broadly that the cross talk between programmed cell death pathways may be important to restrict multiple other microbial pathogens by limiting their intracellular niche. Targeting programmed cell death pathways for therapeutic intervention needs to be carefully considered, as inhibitors may result in alternative and unfavorable forms of cell death during infection. This functional redundancy of cell death pathways also has important implications for treating other diseases ranging from cancer to autoimmunity. Despite the difficulty in targeting these highly interconnected cell death pathways, understanding the mechanisms by which these pathways are coordinated may identify new therapeutic targets which could benefit patients with acute or chronic infections that are currently difficult to treat.

10.1128/mBio.01059-21.4 FIG S4 Graphical model for B. thailandensis infection in BMDMs. Representative infection time-course of B. thailandensis infection in WT bone marrow-derived macrophages (BMDMs), BMDMs deficient in key components of pyroptosis, or BMDMs deficient in key components of PANoptosis (pyroptosis, apoptosis, necroptosis). Download FIG S4, PDF file, 0.05 MB. Copyright © 2021 Place et al. 2021 Place et al. https://creativecommons.org/licenses/by/4.0/ This content is distributed under the terms of the Creative Commons Attribution 4.0 International license.

## MATERIALS AND METHODS

### Mice.

Wild type (C57BL6/J), *Casp1/11*^−/−^ (19), *Gsdmd*^−/−^ (55), *Ripk3*^−/−^ (73), *Casp8/Ripk3*^−/−^ (74), *Mlkl*^−/−^ (75), *Gsdmd/Mlkl*^−/−^ (43), *Casp8/Ripk3/Casp1/11*^−/−^ (generated by crossing *Casp8/Ripk3*^−/−^ and *Casp1/11*^−/−^) (35, 43), or *Casp8/Ripk3/Ripk1*^−/−^ (generated by crossing *Casp8/Ripk3*^−/−^ and *Ripk1^+/−^*) (76) mice have been described previously. Male and female mice between 6 and 8 weeks old were used in this study. Mice were bred at St. Jude Children’s Research Hospital, and studies were conducted under protocols approved by St. Jude Children’s Research Hospital Institutional Committee on the Use and Care of Animals.

### Bone marrow-derived macrophage cultures.

Primary bone marrow-derived macrophages (BMDMs) were grown for 6 days in Iscove’s modified Dulbecco’s medium (IMDM) (12440053, Thermo Fisher Scientific, Waltham, MA) supplemented with 10% fetal bovine serum (FBS) (TMS-013-B, Millipore, Billerica, MA or S1620, BioWest, Riverside, MO), 30% L929-conditioned medium, and 1× penicillin-streptomycin (15070063, Thermo Fisher Scientific). BMDMs were seeded at a concentration of 1 × 10^6^ cells onto 12-well plates or 1 × 10^5^ cells onto 96-well plates and incubated overnight. Cells were washed and cultured in antibiotic-free Dulbecco’s modified Eagle medium (DMEM) (11995065, Thermo Fisher Scientific, Waltham, MA) with 10% FBS before stimulations or infections.

### Bacterial culture.

B. thailandensis strain E264 (green fluorescent protein [GFP]^+^) and B. thailandensis *vgrG5*Δ*CTD* (vgrG5 lacking the C-terminal domain required for fusion activity) (65), provided by Joseph Mougous (University of Washington, Seattle, WA), were cultured in LB broth (3002-031, MP Biomedicals, Santa Ana, CA) overnight and subcultured into fresh LB medium for 3 h at 37°C to generate log-phase cultures.

### Mouse infections.

B. thailandensis was grown as described above. For mouse infection experiments, quantified frozen aliquots of B. thailandensis were diluted in phosphate-buffered saline (PBS) before infection to inoculate 5 × 10^4^ or 5 × 10^2^ bacteria per mouse. Mice were anesthetized with isoflurane and administered B. thailandensis in a 50 μl PBS suspension via the nares. After 2 days of infection, lungs were harvested for histology (tissues fixed in formalin and processed by the St. Jude Children’s Research Hospital Veterinary Pathology Core) or CFU analysis. Histological scoring was performed by a board-certified veterinary pathologist (author P.V.) and assigned a semiquantitative score based on the severity grades 0 = within normal limits; 1 = minimal: rare or inconspicuous lesions; 2 = mild: multifocal or small, focal, or widely separated, but conspicuous lesions; 3 = moderate: multifocal, prominent lesions; 4 = marked: extensive to coalescing lesions or areas of inflammation with some loss of structure; 5 = severe: diffuse lesion with effacement of normal structure. Severity grades were converted to semiquantitative scores with the following criteria: 0 = 0; 1 = 1; 1.5 = 8; 2 = 15; 2.5 = 25; 3 = 40; 3.5 = 60; 4 = 80; 4.5 = 90; 5 = 100. CFU were determined by homogenizing tissue in PBS in Lysing Matrix C tubes on a FastPrep homogenizer (1169120050, MP Biomedicals) and plating on LB agar plates, then incubated overnight at 37°C.

### Bone marrow-derived macrophage stimulations.

BMDMs were differentiated as described above. Prior to stimulation, cells were washed with PBS, and PBS was replaced with fresh antibiotic-free DMEM containing 10% FBS. B. thailandensis (MOI 5) was pelleted onto cells at 300 × *g* for 5 min; cells were washed after 1 h, incubated with DMEM containing 1,000 μg/ml kanamycin for 1 h to kill remaining extracellular B. thailandensis, washed, and finally incubated in DMEM containing 250 μg/ml kanamycin for the remainder of the experiment to restrict extracellular growth of B. thailandensis. After the final wash, SYTOX Green (25 nM, S7020, Thermo Fisher Scientific) or propidium iodide (PI) (250 ng/ml, P3566, Thermo Fisher Scientific) was added to media for IncuCyte experiments. To determine intracellular CFU counts, cells were washed with PBS, lysed with PBS containing 0.01% Triton X-100, serially diluted in PBS, and plated on LB agar plates, then incubated at 37°C.

### Microscopy methods.

For fluorescence microscopy experiments, BMDMs (0.5 × 10^6^) were seeded in a 4-chamber μ-slide (80426, Ibidi). Chambers were infected as described above. Wells were aspirated and fixed in 4% paraformaldehyde (PFA), washed with PBS, permeabilized with 0.1% Triton X-100, and stained with phalloidin-iFluor647 (ab176759, Abcam), and DAPI (4′,6-diamidino-2-phenylindole). For confocal images, a Nikon C2 microscope was used as previously described (59). Quantitative cell death measurements by SYTOX Green or PI uptake counts were collected hourly on an IncuCyte S3 system (Essen BioScience). Within each replicate well (on a 96-well plate), nine images were collected, and cell death was quantified using the Basic Analyzer software of the IncuCyte S3 and exported as “object count per well,” which extrapolates the “object count” from the average count across images to the total well area. At least four replicate wells were included for each experiment, and these replicate wells were used to determine statistical significance between treatment conditions.

### Immunoblotting analysis.

For signaling blots, the supernatant was removed, and cells were lysed in radioimmunoprecipitation assay (RIPA) buffer containing protease and phosphatase inhibitors plus 4× Laemmli sample buffer. Caspase and GSDMD cleavage were measured from the combined cell lysate and supernatants. Proteins were separated via SDS-PAGE with 8 to 12% polyacrylamide gels, transferred to polyvinylidene difluoride (PVDF) membranes (IPVH00010, Millipore), and blocked with 5% nonfat dry milk. Primary antibodies against caspase-1 (AG-20B-0042-C100, Adipogen), caspase-3/cleaved-caspase-3 (9662 and 9661, CST), caspase-7/cleaved-caspase-7 (9492 and 9491, CST), caspase-8/cleaved-caspase-8 (4927, CST and AG-20T-0138-C100, Adipogen), caspase-9 (9504, CST), GSDMD (ab209845, Abcam), p-RIPK3 (91702, CST), RIPK3 (2283, ProSci), p-MLKL/MLKL (37333 and 37705, CST), and β-actin (4970, CST) were incubated overnight at 4°C followed by appropriate secondary antibodies conjugated with horseradish peroxidase (HRP) incubated for 1 h at room temperature (Jackson ImmunoResearch, West Grove, PA). Membranes were visualized using Luminata Forte Chemiluminescence substrate (WBLUF0500, Millipore) or SuperSignal West Femto substrate (34096, Thermo Fisher Scientific) on a Bio-Rad ChemiDoc.

### Quantification and statistical analysis.

GraphPad Prism 6.0 software was used for data analysis. Data are shown as mean ± standard error of the mean (SEM). Statistical significance was determined by one-way or two-way ANOVA with the Dunn’s, Tukey, or Holm-Sidak multiple-comparison test. The specific statistical testing for each experiment is indicated in the figure legends. Survival curves were compared using the log-rank test. *P* < 0.05 was considered statistically significant.

### Data availability.

Further information and requests for resources and reagents should be directed to and will be fulfilled by the corresponding author, Thirumala-Devi Kanneganti (thirumala-devi.kanneganti@stjude.org).

## References

[1] Sousa SA, Ramos CG, Leitão JH. 2011. Burkholderia cepacia complex: emerging multihost pathogens equipped with a wide range of virulence factors and determinants. Int J Microbiol 2011:1–9. doi:10.1155/2011/607575. PMC2929507 20811541

[2] Van Zandt KE, Greer MT, Gelhaus HC. 2013. Glanders: an overview of infection in humans. Orphanet J Rare Dis 8:131. doi:10.1186/1750-1172-8-131. 24004906 PMC3766238

[3] Adler B. 2011. Strategies for intracellular survival of Burkholderia pseudomallei. Front Microbiol 2:170. doi:10.3389/fmicb.2011.00170. 22007185 PMC3159172

[4] Benanti EL, Nguyen CM, Welch MD. 2015. Virulent Burkholderia species mimic host actin polymerases to drive actin-based motility. Cell 161:348–360. doi:10.1016/j.cell.2015.02.044. 25860613 PMC4393530

[5] Haraga A, West TE, Brittnacher MJ, Skerrett SJ, Miller SI. 2008. Burkholderia thailandensis as a model system for the study of the virulence-associated type III secretion system of Burkholderia pseudomallei. Infect Immun 76:5402–5411. doi:10.1128/IAI.00626-08. 18779342 PMC2573339

[6] Galyov EE, Brett PJ, DeShazer D. 2010. Molecular insights into Burkholderia pseudomallei and Burkholderia mallei pathogenesis. Annu Rev Microbiol 64:495–517. doi:10.1146/annurev.micro.112408.134030. 20528691

[7] Wiersinga WJ, van der Poll T. 2009. Immunity to Burkholderia pseudomallei. Curr Opin Infect Dis 22:102–108. doi:10.1097/QCO.0b013e328322e727. 19276877

[8] Fuchs Y, Steller H. 2011. Programmed cell death in animal development and disease. Cell 147:742–758. doi:10.1016/j.cell.2011.10.033. 22078876 PMC4511103

[9] Cookson BT, Brennan MA. 2001. Pro-inflammatory programmed cell death. Trends Microbiol 9:113–114. doi:10.1016/s0966-842x(00)01936-3. 11303500

[10] Agard NJ, Maltby D, Wells JA. 2010. Inflammatory stimuli regulate caspase substrate profiles. Mol Cell Proteomics 9:880–893. doi:10.1074/mcp.M900528-MCP200. 20173201 PMC2871421

[11] He W-T, Wan H, Hu L, Chen P, Wang X, Huang Z, Yang Z-H, Zhong C-Q, Han J. 2015. Gasdermin D is an executor of pyroptosis and required for interleukin-1β secretion. Cell Res 25:1285–1298. doi:10.1038/cr.2015.139. 26611636 PMC4670995

[12] Kayagaki N, Stowe IB, Lee BL, O'Rourke K, Anderson K, Warming S, Cuellar T, Haley B, Roose-Girma M, Phung QT, Liu PS, Lill JR, Li H, Wu J, Kummerfeld S, Zhang J, Lee WP, Snipas SJ, Salvesen GS, Morris LX, Fitzgerald L, Zhang Y, Bertram EM, Goodnow CC, Dixit VM. 2015. Caspase-11 cleaves gasdermin D for non-canonical inflammasome signalling. Nature 526:666–671. doi:10.1038/nature15541. 26375259

[13] Li P, Allen H, Banerjee S, Franklin S, Herzog L, Johnston C, McDowell J, Paskind M, Rodman L, Salfeld J. 1995. Mice deficient in IL-1 beta-converting enzyme are defective in production of mature IL-1 beta and resistant to endotoxic shock. Cell 80:401–411. doi:10.1016/0092-8674(95)90490-5. 7859282

[14] Martinon F, Burns K, Tschopp J. 2002. The inflammasome. Mol Cell 10:417–426. doi:10.1016/s1097-2765(02)00599-3. 12191486

[15] Place DE, Kanneganti T-D. 2019. Cell death-mediated cytokine release and its therapeutic implications. J Experimental Medicine 216:e20181892. doi:10.1084/jem.20181892. PMC6605758 31186281

[16] Shi J, Zhao Y, Wang K, Shi X, Wang Y, Huang H, Zhuang Y, Cai T, Wang F, Shao F. 2015. Cleavage of GSDMD by inflammatory caspases determines pyroptotic cell death. Nature 526:660–665. doi:10.1038/nature15514. 26375003

[17] Thornberry NA, Bull HG, Calaycay JR, Chapman KT, Howard AD, Kostura MJ, Miller DK, Molineaux SM, Weidner JR, Aunins J. 1992. A novel heterodimeric cysteine protease is required for interleukin-1 beta processing in monocytes. Nature 356:768–774. doi:10.1038/356768a0. 1574116

[18] Hagar JA, Powell DA, Aachoui Y, Ernst RK, Miao EA. 2013. Cytoplasmic LPS activates caspase-11: implications in TLR4-independent endotoxic shock. Science 341:1250–1253. doi:10.1126/science.1240988. 24031018 PMC3931427

[19] Kayagaki N, Warming S, Lamkanfi M, Vande Walle L, Louie S, Dong J, Newton K, Qu Y, Liu J, Heldens S, Zhang J, Lee WP, Roose-Girma M, Dixit VM. 2011. Non-canonical inflammasome activation targets caspase-11. Nature 479:117–121. doi:10.1038/nature10558. 22002608

[20] Kayagaki N, Wong MT, Stowe IB, Ramani SR, Gonzalez LC, Akashi-Takamura S, Miyake K, Zhang J, Lee WP, Muszyński A, Forsberg LS, Carlson RW, Dixit VM. 2013. Noncanonical inflammasome activation by intracellular LPS independent of TLR4. Science 341:1246–1249. doi:10.1126/science.1240248. 23887873

[21] Shi J, Zhao Y, Wang Y, Gao W, Ding J, Li P, Hu L, Shao F. 2014. Inflammatory caspases are innate immune receptors for intracellular LPS. Nature 514:187–192. doi:10.1038/nature13683. 25119034

[22] Kaiser WJ, Sridharan H, Huang C, Mandal P, Upton JW, Gough PJ, Sehon CA, Marquis RW, Bertin J, Mocarski ES. 2013. Toll-like receptor 3-mediated necrosis via TRIF, RIP3, and MLKL. J Biol Chem 288:31268–31279. doi:10.1074/jbc.M113.462341. 24019532 PMC3829437

[23] Kuriakose T, Man SM, Malireddi RKS, Karki R, Kesavardhana S, Place DE, Neale G, Vogel P, Kanneganti T-D. 2016. ZBP1/DAI is an innate sensor of influenza virus triggering the NLRP3 inflammasome and programmed cell death pathways. Sci Immunol 1:aag2045. doi:10.1126/sciimmunol.aag2045. 27917412 PMC5131924

[24] Newton K, Wickliffe KE, Maltzman A, Dugger DL, Strasser A, Pham VC, Lill JR, Roose-Girma M, Warming S, Solon M, Ngu H, Webster JD, Dixit VM. 2016. RIPK1 inhibits ZBP1-driven necroptosis during development. Nature 540:129–133. doi:10.1038/nature20559. 27819682

[25] Sun L, Wang H, Wang Z, He S, Chen S, Liao D, Wang L, Yan J, Liu W, Lei X, Wang X. 2012. Mixed lineage kinase domain-like protein mediates necrosis signaling downstream of RIP3 kinase. Cell 148:213–227. doi:10.1016/j.cell.2011.11.031. 22265413

[26] Zhao J, Jitkaew S, Cai Z, Choksi S, Li Q, Luo J, Liu Z-G. 2012. Mixed lineage kinase domain-like is a key receptor interacting protein 3 downstream component of TNF-induced necrosis. Proc Natl Acad Sci U S A 109:5322–5327. doi:10.1073/pnas.1200012109. 22421439 PMC3325682

[27] Cho YS, Challa S, Moquin D, Genga R, Ray TD, Guildford M, Chan FK-M. 2009. Phosphorylation-driven assembly of the RIP1-RIP3 complex regulates programmed necrosis and virus-induced inflammation. Cell 137:1112–1123. doi:10.1016/j.cell.2009.05.037. 19524513 PMC2727676

[28] He S, Wang L, Miao L, Wang T, Du F, Zhao L, Wang X. 2009. Receptor interacting protein kinase-3 determines cellular necrotic response to TNF-alpha. Cell 137:1100–1111. doi:10.1016/j.cell.2009.05.021. 19524512

[29] Newton K, Dugger DL, Wickliffe KE, Kapoor N, Almagro M.d, Vucic D, Komuves L, Ferrando RE, French DM, Webster J, Roose-Girma M, Warming S, Dixit VM. 2014. Activity of protein kinase RIPK3 determines whether cells die by necroptosis or apoptosis. Science 343:1357–1360. doi:10.1126/science.1249361. 24557836

[30] Sun X, Lee J, Navas T, Baldwin DT, Stewart TA, Dixit VM. 1999. RIP3, a novel apoptosis-inducing kinase. J Biol Chem 274:16871–16875. doi:10.1074/jbc.274.24.16871. 10358032

[31] Zhang D-W, Shao J, Lin J, Zhang N, Lu B-J, Lin S-C, Dong M-Q, Han J. 2009. RIP3, an energy metabolism regulator that switches TNF-induced cell death from apoptosis to necrosis. Science 325:332–336. doi:10.1126/science.1172308. 19498109

[32] Van Opdenbosch N, Lamkanfi M. 2019. Caspases in cell death, inflammation, and disease. Immunity 50:1352–1364. doi:10.1016/j.immuni.2019.05.020. 31216460 PMC6611727

[33] Arandjelovic S, Ravichandran KS. 2015. Phagocytosis of apoptotic cells in homeostasis. 9. Nat Immunol 16:907–917. doi:10.1038/ni.3253. 26287597 PMC4826466

[34] Gurung P, Anand PK, Malireddi RKS, Walle LV, Opdenbosch NV, Dillon CP, Weinlich R, Green DR, Lamkanfi M, Kanneganti T-D. 2014. FADD and caspase-8 mediate priming and activation of the canonical and noncanonical Nlrp3 inflammasomes. J Immunol 192:1835–1846. doi:10.4049/jimmunol.1302839. 24453255 PMC3933570

[35] Gurung P, Burton A, Kanneganti T-D. 2016. NLRP3 inflammasome plays a redundant role with caspase 8 to promote IL-1β-mediated osteomyelitis. Proc Natl Acad Sci U S A 113:4452–4457. doi:10.1073/pnas.1601636113. 27071119 PMC4843439

[36] Lamkanfi M, Kanneganti T-D, Van Damme P, Vanden Berghe T, Vanoverberghe I, Vandekerckhove J, Vandenabeele P, Gevaert K, Núñez G. 2008. Targeted peptidecentric proteomics reveals caspase-7 as a substrate of the caspase-1 inflammasomes. Mol Cell Proteomics 7:2350–2363. doi:10.1074/mcp.M800132-MCP200. 18667412 PMC2596343

[37] Lukens JR, Gross JM, Calabrese C, Iwakura Y, Lamkanfi M, Vogel P, Kanneganti T-D. 2014. Critical role for inflammasome-independent IL-1β production in osteomyelitis. Proc Natl Acad Sci U S A 111:1066–1071. doi:10.1073/pnas.1318688111. 24395792 PMC3903206

[38] Yu J, Li S, Qi J, Chen Z, Wu Y, Guo J, Wang K, Sun X, Zheng J. 2019. Cleavage of GSDME by caspase-3 determines lobaplatin-induced pyroptosis in colon cancer cells. Cell Death Dis 10:193. doi:10.1038/s41419-019-1441-4. 30804337 PMC6389936

[39] Conos SA, Chen KW, De Nardo D, Hara H, Whitehead L, Núñez G, Masters SL, Murphy JM, Schroder K, Vaux DL, Lawlor KE, Lindqvist LM, Vince JE. 2017. Active MLKL triggers the NLRP3 inflammasome in a cell-intrinsic manner. Proc Natl Acad Sci U S A 114:E961–E969. doi:10.1073/pnas.1613305114. 28096356 PMC5307433

[40] Place DE, Lee S, Kanneganti T-D. 2021. PANoptosis in microbial infection. Curr Opin Microbiol 59:42–49. doi:10.1016/j.mib.2020.07.012. 32829024 PMC7438227

[41] Samir P, Malireddi RKS, Kanneganti T-D. 2020. The PANoptosome: a deadly protein complex driving pyroptosis, apoptosis, and necroptosis (PANoptosis). Front Cell Infect Microbiol 10:238. doi:10.3389/fcimb.2020.00238. 32582562 PMC7283380

[42] Malireddi RKS, Kesavardhana S, Kanneganti T-D. 2019. ZBP1 and TAK1: master regulators of NLRP3 inflammasome/pyroptosis, apoptosis, and necroptosis (PAN-optosis). Front Cell Infect Microbiol 9:406. doi:10.3389/fcimb.2019.00406. 31850239 PMC6902032

[43] Christgen S, Zheng M, Kesavardhana S, Karki R, Malireddi RKS, Banoth B, Place DE, Briard B, Sharma BR, Tuladhar S, Samir P, Burton A, Kanneganti T-D. 2020. Identification of the PANoptosome: a molecular platform triggering cell death. Front Cell Infect Microbiol 10:237. doi:10.3389/fcimb.2020.00237. 32547960 PMC7274033

[44] Zheng M, Karki R, Vogel P, Kanneganti T-D. 2020. Caspase-6 is a key regulator of innate immunity, inflammasome activation, and host defense. Cell 181:674–687. doi:10.1016/j.cell.2020.03.040. 32298652 PMC7425208

[45] Malireddi RKS, Gurung P, Kesavardhana S, Samir P, Burton A, Mummareddy H, Vogel P, Pelletier S, Burgula S, Kanneganti T-D. 2020. Innate immune priming in the absence of TAK1 drives RIPK1 kinase activity-independent pyroptosis, apoptosis, necroptosis, and inflammatory disease. J Exp Med 217:jem20191644. doi:10.1084/jem.20191644. PMC7062518 31869420

[46] Malireddi RKS, Kesavardhana S, Karki R, Kancharana B, Burton AR, Kanneganti T-D. 2020. RIPK1 distinctly regulates Yersinia-induced inflammatory cell death, PANoptosis. Immunohorizons 4:789–796. doi:10.4049/immunohorizons.2000097. 33310881 PMC7906112

[47] Karki R, Sharma BR, Lee E, Banoth B, Malireddi RKS, Samir P, Tuladhar S, Mummareddy H, Burton AR, Vogel P, Kanneganti T-D. 2020. Interferon regulatory factor 1 regulates PANoptosis to prevent colorectal cancer. JCI Insight 5:e136720. doi:10.1172/jci.insight.136720. PMC7406299 32554929

[48] Malireddi RKS, Gurung P, Mavuluri J, Dasari TK, Klco JM, Chi H, Kanneganti T-D. 2018. TAK1 restricts spontaneous NLRP3 activation and cell death to control myeloid proliferation. J Exp Med 215:1023–1034. doi:10.1084/jem.20171922. 29500178 PMC5881469

[49] Zheng M, Williams EP, Malireddi RKS, Karki R, Banoth B, Burton A, Webby R, Channappanavar R, Jonsson CB, Kanneganti T-D. 2020. Impaired NLRP3 inflammasome activation/pyroptosis leads to robust inflammatory cell death via caspase-8/RIPK3 during coronavirus infection. J Biol Chem 295:14040–14052. doi:10.1074/jbc.RA120.015036. 32763970 PMC7549031

[50] Banoth B, Tuladhar S, Karki R, Sharma BR, Briard B, Kesavardhana S, Burton A, Kanneganti T-D. 2020. ZBP1 promotes fungi-induced inflammasome activation and pyroptosis, apoptosis, and necroptosis (PANoptosis). J Biol Chem 295:18276–18283. doi:10.1074/jbc.RA120.015924. 33109609 PMC7939383

[51] Karki R, Sharma BR, Tuladhar S, Williams EP, Zalduondo L, Samir P, Zheng M, Sundaram B, Banoth B, Malireddi RKS, Schreiner P, Neale G, Vogel P, Webby R, Jonsson CB, Kanneganti T-D. 2021. Synergism of TNF-α and IFN-γ triggers inflammatory cell death, tissue damage, and mortality in SARS-CoV-2 infection and cytokine shock syndromes. Cell 184:149–168. doi:10.1016/j.cell.2020.11.025. 33278357 PMC7674074

[52] Aachoui Y, Kajiwara Y, Leaf IA, Mao D, Ting JP-Y, Coers J, Aderem A, Buxbaum JD, Miao EA. 2015. Canonical inflammasomes drive IFN-γ to prime caspase-11 in defense against a cytosol-invasive bacterium. Cell Host Microbe 18:320–332. doi:10.1016/j.chom.2015.07.016. 26320999 PMC4567510

[53] Bast A, Krause K, Schmidt IHE, Pudla M, Brakopp S, Hopf V, Breitbach K, Steinmetz I. 2014. Caspase-1-dependent and -independent cell death pathways in Burkholderia pseudomallei infection of macrophages. PLoS Pathog 10:e1003986. doi:10.1371/journal.ppat.1003986. 24626296 PMC3953413

[54] Ceballos-Olvera I, Sahoo M, Miller MA, Del Barrio L, Re F. 2011. Inflammasome-dependent pyroptosis and IL-18 protect against Burkholderia pseudomallei lung infection while IL-1β is deleterious. PLoS Pathog 7:e1002452. doi:10.1371/journal.ppat.1002452. 22241982 PMC3248555

[55] Karki R, Lee E, Place D, Samir P, Mavuluri J, Sharma BR, Balakrishnan A, Malireddi RKS, Geiger R, Zhu Q, Neale G, Kanneganti T-D. 2018. IRF8 regulates transcription of Naips for NLRC4 inflammasome activation. Cell 173:920–933. doi:10.1016/j.cell.2018.02.055. 29576451 PMC5935577

[56] Place DE, Samir P, Karki R, Briard B, Vogel P, Kanneganti T-D. 2018. ASK family kinases are required for optimal NLRP3 inflammasome priming. Am J Pathol 188:1021–1030. doi:10.1016/j.ajpath.2017.12.006. 29353059 PMC6436110

[57] Sahoo M, Lantier L, Re F. 2016. Role of canonical and non-canonical inflammasomes during Burkholderia infection. Curr Top Microbiol Immunol 397:199–214. doi:10.1007/978-3-319-41171-2_10. 27460811

[58] Wang J, Deobald K, Re F. 2019. Gasdermin D protects from melioidosis through pyroptosis and direct killing of bacteria. J Immunol 202:3468–3473. doi:10.4049/jimmunol.1900045. 31036765 PMC6548608

[59] Place DE, Briard B, Samir P, Karki R, Bhattacharya A, Guy CS, Peters JL, Frase S, Vogel P, Neale G, Yamamoto M, Kanneganti T-D. 2020. Interferon inducible GBPs restrict Burkholderia thailandensis motility induced cell-cell fusion. PLoS Pathog 16:e1008364. doi:10.1371/journal.ppat.1008364. 32150572 PMC7082077

[60] Wang J, Sahoo M, Lantier L, Warawa J, Cordero H, Deobald K, Re F. 2018. Caspase-11-dependent pyroptosis of lung epithelial cells protects from melioidosis while caspase-1 mediates macrophage pyroptosis and production of IL-18. PLoS Pathog 14:e1007105. doi:10.1371/journal.ppat.1007105. 29791511 PMC5988316

[61] Alvarez-Diaz S, Dillon CP, Lalaoui N, Tanzer MC, Rodriguez DA, Lin A, Lebois M, Hakem R, Josefsson EC, O'Reilly LA, Silke J, Alexander WS, Green DR, Strasser A. 2016. The pseudokinase MLKL and the kinase RIPK3 have distinct roles in autoimmune disease caused by loss of death receptor induced apoptosis. Immunity 45:513–526. doi:10.1016/j.immuni.2016.07.016. 27523270 PMC5040700

[62] Kaiser WJ, Upton JW, Long AB, Livingston-Rosanoff D, Daley-Bauer LP, Hakem R, Caspary T, Mocarski ES. 2011. RIP3 mediates the embryonic lethality of caspase-8-deficient mice. Nature 471:368–372. doi:10.1038/nature09857. 21368762 PMC3060292

[63] French CT, Toesca IJ, Wu T-H, Teslaa T, Beaty SM, Wong W, Liu M, Schröder I, Chiou P-Y, Teitell MA, Miller JF. 2011. Dissection of the Burkholderia intracellular life cycle using a photothermal nanoblade. Proc Natl Acad Sci U S A 108:12095–12100. doi:10.1073/pnas.1107183108. 21730143 PMC3141958

[64] Kespichayawattana W, Rattanachetkul S, Wanun T, Utaisincharoen P, Sirisinha S. 2000. Burkholderia pseudomallei induces cell fusion and actin-associated membrane protrusion: a possible mechanism for cell-to-cell spreading. Infect Immun 68:5377–5384. doi:10.1128/iai.68.9.5377-5384.2000. 10948167 PMC101801

[65] Schwarz S, Singh P, Robertson JD, LeRoux M, Skerrett SJ, Goodlett DR, West TE, Mougous JD. 2014. VgrG-5 is a Burkholderia type VI secretion system-exported protein required for multinucleated giant cell formation and virulence. Infect Immun 82:1445–1452. doi:10.1128/IAI.01368-13. 24452686 PMC3993412

[66] Toesca IJ, French CT, Miller JF. 2014. The Type VI secretion system spike protein VgrG5 mediates membrane fusion during intercellular spread by pseudomallei group Burkholderia species. Infect Immun 82:1436–1444. doi:10.1128/IAI.01367-13. 24421040 PMC3993413

[67] Schwarz S, West TE, Boyer F, Chiang W-C, Carl MA, Hood RD, Rohmer L, Tolker-Nielsen T, Skerrett SJ, Mougous JD. 2010. Burkholderia type VI secretion systems have distinct roles in eukaryotic and bacterial cell interactions. PLoS Pathog 6:e1001068. doi:10.1371/journal.ppat.1001068. 20865170 PMC2928800

[68] Kesavardhana S, Kuriakose T, Guy CS, Samir P, Malireddi RKS, Mishra A, Kanneganti T-D. 2017. ZBP1/DAI ubiquitination and sensing of influenza vRNPs activate programmed cell death. J Exp Med 214:2217–2229. doi:10.1084/jem.20170550. 28634194 PMC5551577

[69] Orning P, Weng D, Starheim K, Ratner D, Best Z, Lee B, Brooks A, Xia S, Wu H, Kelliher MA, Berger SB, Gough PJ, Bertin J, Proulx MM, Goguen JD, Kayagaki N, Fitzgerald KA, Lien E. 2018. Pathogen blockade of TAK1 triggers caspase-8-dependent cleavage of gasdermin D and cell death. Science 362:1064–1069. doi:10.1126/science.aau2818. 30361383 PMC6522129

[70] Sarhan J, Liu BC, Muendlein HI, Li P, Nilson R, Tang AY, Rongvaux A, Bunnell SC, Shao F, Green DR, Poltorak A. 2018. Caspase-8 induces cleavage of gasdermin D to elicit pyroptosis during Yersinia infection. Proc Natl Acad Sci U S A 115:E10888–E10897. doi:10.1073/pnas.1809548115. 30381458 PMC6243247

[71] Ashida H, Sasakawa C, Suzuki T. 2020. A unique bacterial tactic to circumvent the cell death crosstalk induced by blockade of caspase-8. EMBO J 39:e104469. doi:10.15252/embj.2020104469. 32657447 PMC7459423

[72] Price EP, Sarovich DS, Mayo M, Tuanyok A, Drees KP, Kaestli M, Beckstrom-Sternberg SM, Babic-Sternberg JS, Kidd TJ, Bell SC, Keim P, Pearson T, Currie BJ. 2013. Within-Host Evolution of Burkholderia pseudomallei over a Twelve-Year Chronic Carriage Infection. mBio 4:e00388-13. doi:10.1128/mBio.00388-13. 23860767 PMC3735121

[73] Newton K, Sun X, Dixit VM. 2004. Kinase RIP3 is dispensable for normal NF-kappa Bs, signaling by the B-cell and T-cell receptors, tumor necrosis factor receptor 1, and Toll-like receptors 2 and 4. Mol Cell Biol 24:1464–1469. doi:10.1128/mcb.24.4.1464-1469.2004. 14749364 PMC344190

[74] Oberst A, Dillon CP, Weinlich R, McCormick LL, Fitzgerald P, Pop C, Hakem R, Salvesen GS, Green DR. 2011. Catalytic activity of the caspase-8-FLIPL complex inhibits RIPK3-dependent necrosis. Nature 471:363–367. doi:10.1038/nature09852. 21368763 PMC3077893

[75] Murphy JM, Czabotar PE, Hildebrand JM, Lucet IS, Zhang J-G, Alvarez-Diaz S, Lewis R, Lalaoui N, Metcalf D, Webb AI, Young SN, Varghese LN, Tannahill GM, Hatchell EC, Majewski IJ, Okamoto T, Dobson RCJ, Hilton DJ, Babon JJ, Nicola NA, Strasser A, Silke J, Alexander WS. 2013. The pseudokinase MLKL mediates necroptosis via a molecular switch mechanism. Immunity 39:443–453. doi:10.1016/j.immuni.2013.06.018. 24012422

[76] Dillon CP, Weinlich R, Rodriguez DA, Cripps JG, Quarato G, Gurung P, Verbist KC, Brewer TL, Llambi F, Gong Y-N, Janke LJ, Kelliher MA, Kanneganti T-D, Green DR. 2014. RIPK1 blocks early postnatal lethality mediated by caspase-8 and RIPK3. Cell 157:1189–1202. doi:10.1016/j.cell.2014.04.018. 24813850 PMC4068710

