# Supplementary figures and images for: Hierarchical Cell Death Program Disrupts the Intracellular Niche Required for Burkholderia thailandensis Pathogenesis
Source: mBio. 2021 Jun 22;12(3):e01059-21. doi: 10.1128/mBio.01059-21 (PMC8262894; doi:10.1128/mBio.01059-21)

**A**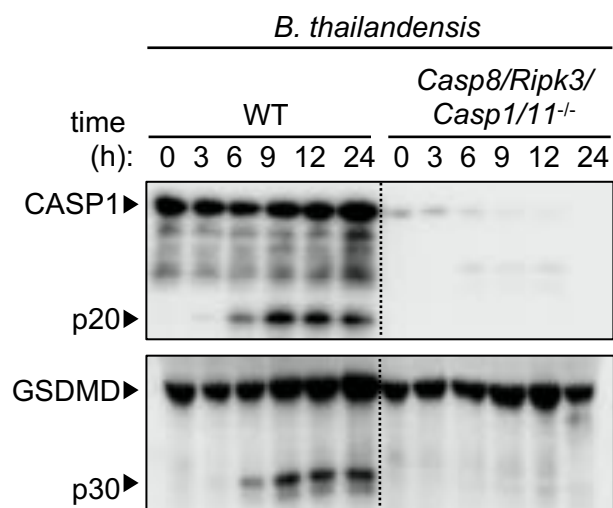**B**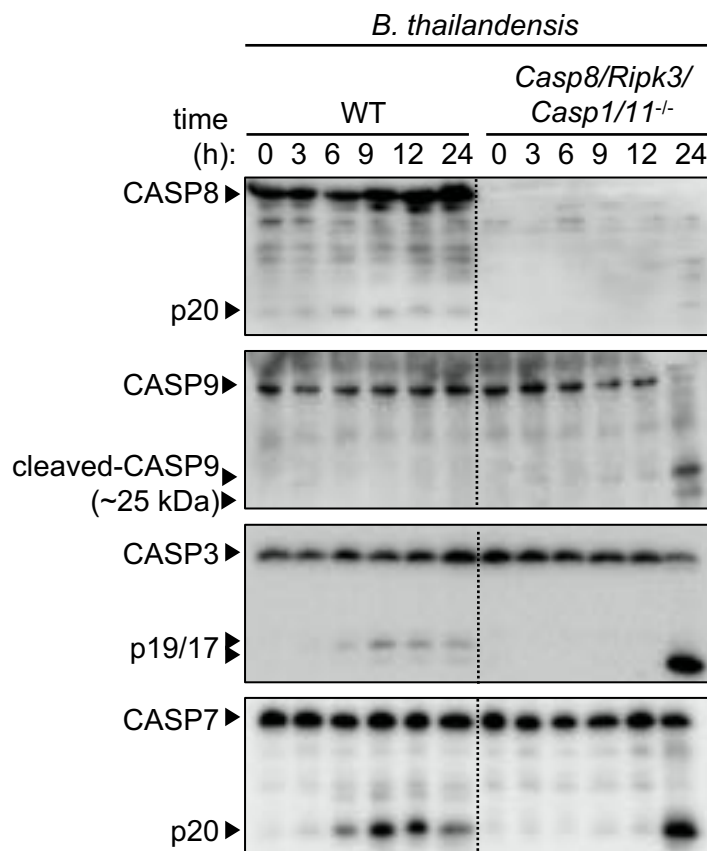**C**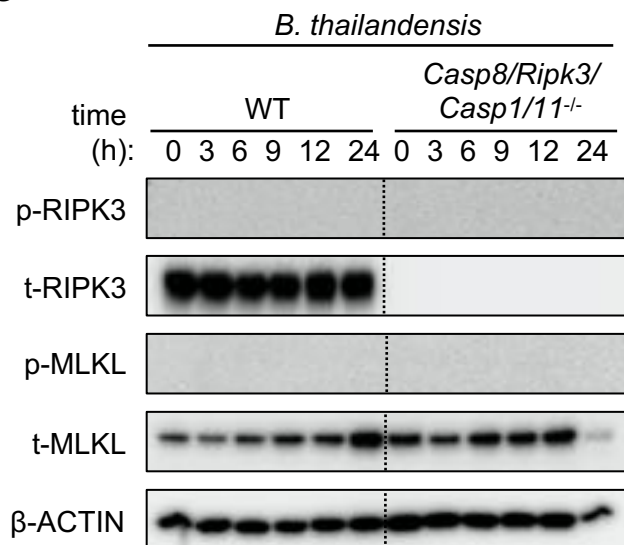

Supplement: FIG S1 [file mbio.01059-21-sf001.pdf]

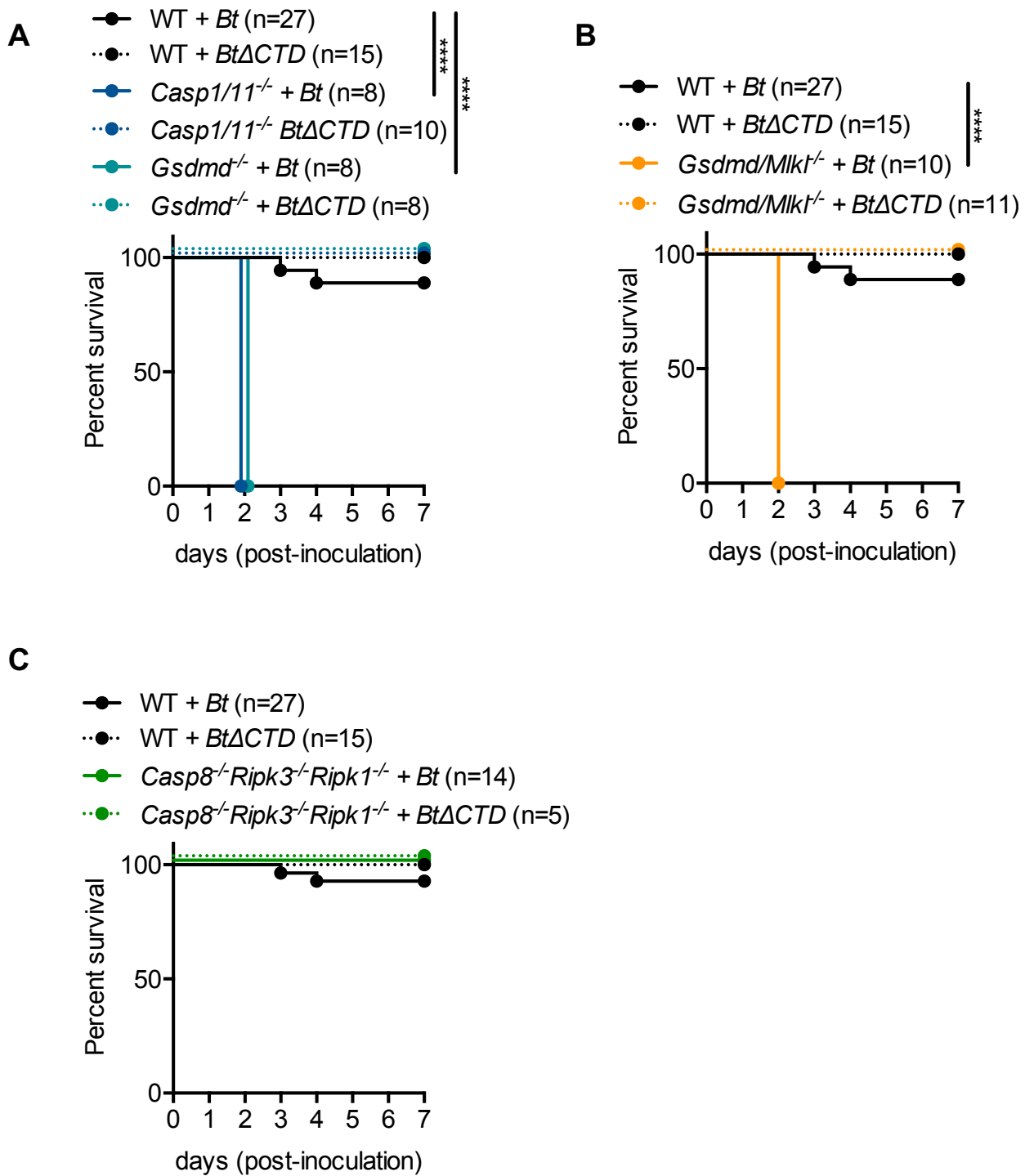

Supplemental Figure 2

Supplement: FIG S2 [file mbio.01059-21-sf002.pdf]

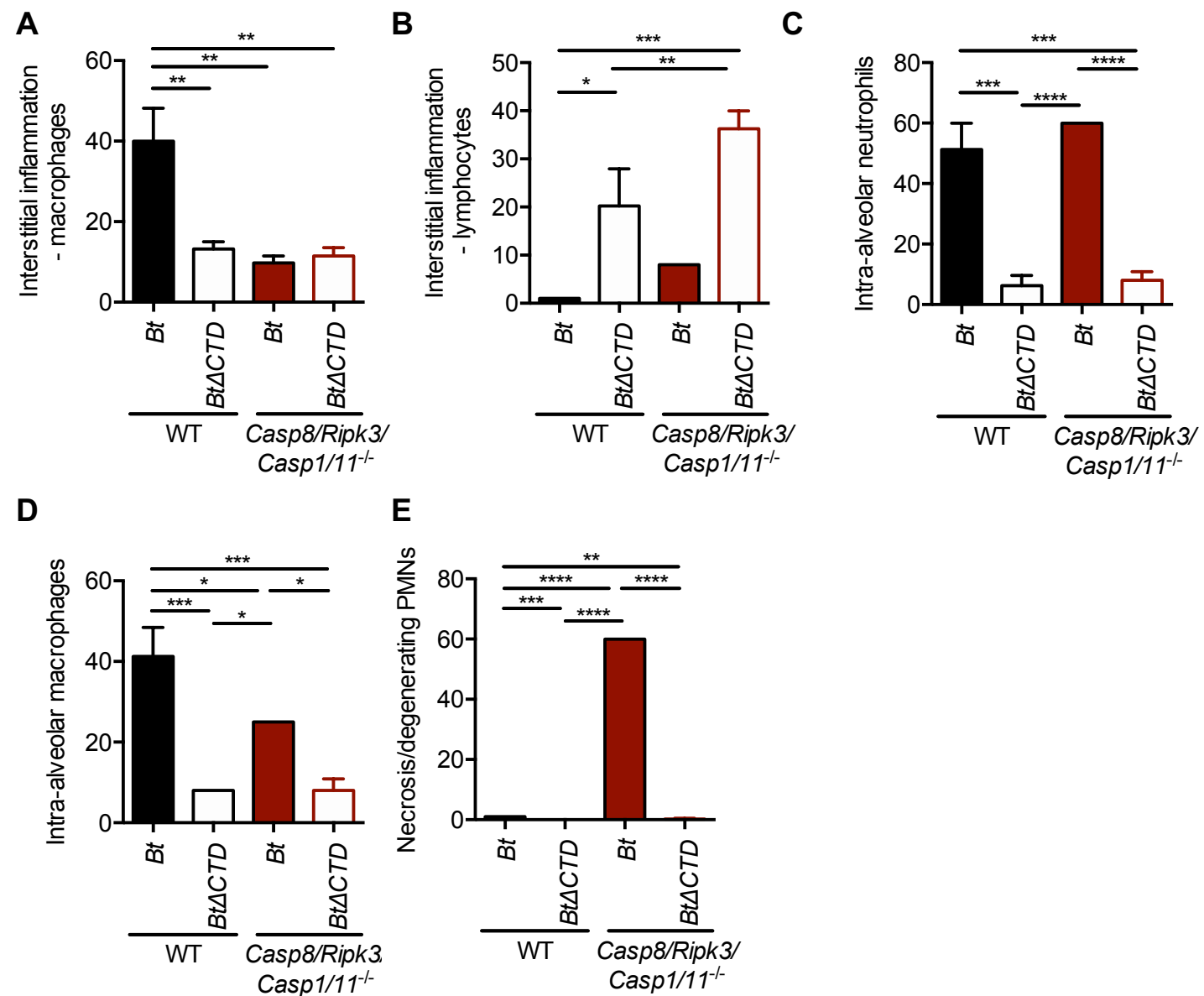

Supplemental Figure 3

Supplement: FIG S3 [file mbio.01059-21-sf003.pdf]

*B. thailandensis*

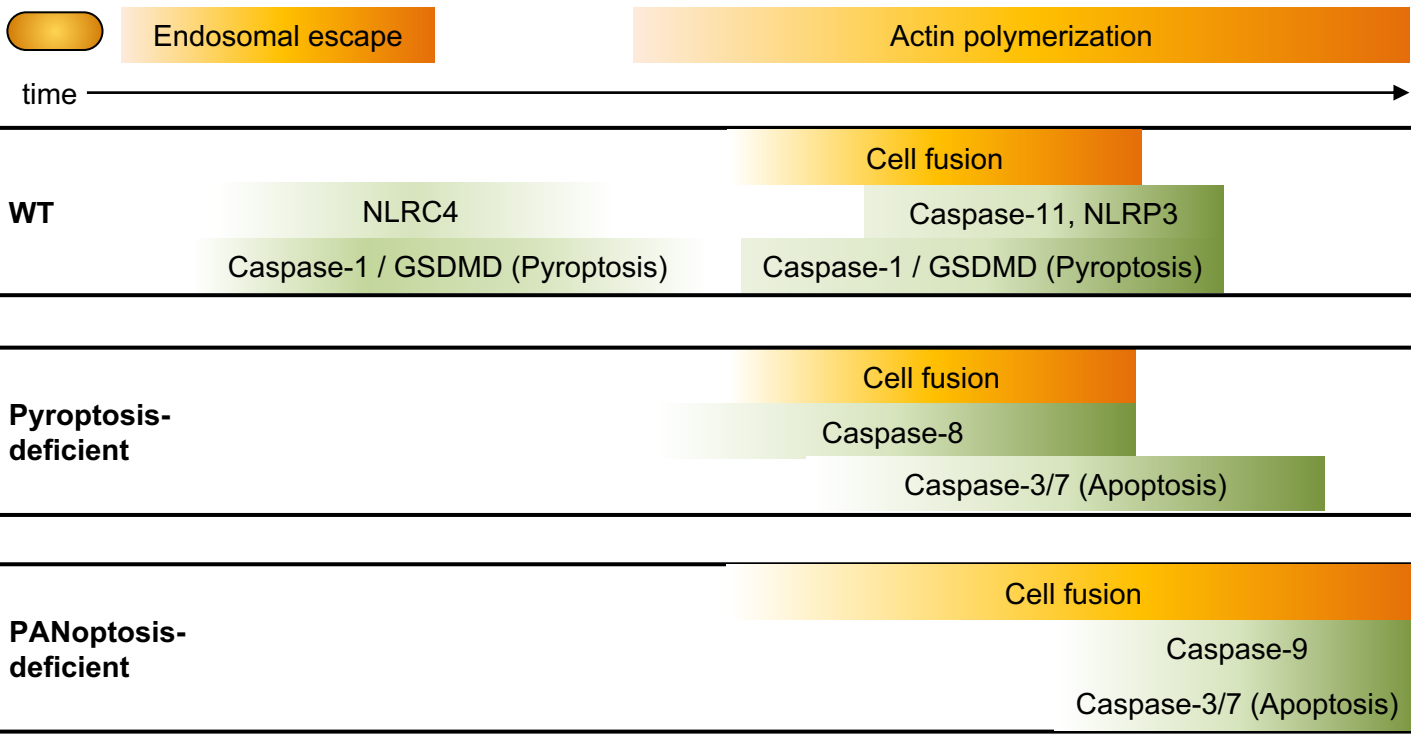

Supplement: FIG S4 [file mbio.01059-21-sf004.pdf]
